# Molecular Findings Before Vision Loss in the Streptozotocin-Induced Rat Model of Diabetic Retinopathy

**DOI:** 10.3390/cimb47010028

**Published:** 2025-01-04

**Authors:** Mădălina Moldovan, Roxana-Denisa Capraș, Raluca Paşcalău, Gabriela Adriana Filip

**Affiliations:** 1 Department of Anatomy and Embryology, Iuliu Hatieganu University of Medicine and Pharmacy, 400012 Cluj-Napoca, Romania; moldovan.madalina@elearn.umfcluj.ro (M.M.);; 2 Ophthalmology Clinic, Cluj County Emergency Hospital, 400006 Cluj-Napoca, Romania; 3 Research and Development Institute, Transilvania University of Brasov, 500484 Brasov, Romania

**Keywords:** diabetic retinopathy, rat, oxidative stress, inflammation, microglia

## Abstract

The streptozotocin-induced rat model of diabetic retinopathy presents similarities to the disease observed in humans. After four weeks following the induction of diabetes, the rats experience vision impairment. During this crucial four-week period, significant changes occur, with vascular damage standing out as a clinically significant factor, alongside neovascularization. While redox imbalance, activation of microglia, secretion of pro-inflammatory cytokines, and neuronal cell death are also observed, the latter remains an emerging hypothesis requiring further exploration. This review is a comprehensive and up-to-date chronological depiction of the progression of diabetic retinopathy within the initial four weeks of hyperglycemia, which precede the onset of vision loss. The data are structured in weekly changes. In the first week, oxidative stress triggers the activation of retinal microglia, which produces inflammation, leading to altered neurotransmission. The second week is characterized by leukostasis, which promotes ischemia, while neural degeneration begins and is accompanied by a simultaneous increase in vessel permeability. The progression of redox and inflammatory imbalances characterized the third week. Finally, in the fourth week, significant developments occur as vessels dilate and become tortuous, neovascularization develops, and retinal thickness diminishes, ultimately leading to vision loss. Through this clearly structured outline, this review aims to delineate a framework for the progression of streptozotocin-induced diabetic retinopathy.

## 1. Introduction

Diabetic retinopathy (DR) is a primary contributor to vision impairment [1]—and existing treatments for advanced stages fail to compensate for the lack of timely interventions [2]. Vision loss in diabetes can be classified into three categories. The first is abrupt vision loss caused by the vitreo-retinal complications of proliferative diabetic retinopathy, including vitreous hemorrhage, retinal traction, retinal detachment, and neovascular glaucoma. The second is vision impairment due to diabetes-induced cataracts, which can be effectively treated with surgery. The third category is a slow, progressive decline in vision resulting from neural cell degeneration. While current therapeutic strategies primarily aim to prevent the first type and efficient surgical options exist for the second, the third category remains more subtle and less clinically apparent [3]. Fortunately, recent years have seen advances in the development of early therapies by leveraging hyperglycemic animal models [4].

Out of all preclinical models, the rat one is often employed due to its accessibility, low cost, and ease of handling [4]. In this model, research data have indicated that vision impairments manifest after four weeks of hyperglycemia, being preceded by changes in both structure and function [5]. Therefore, a potential management could involve addressing the pathological alterations made within this four-week timeframe. By developing therapies that intervene before a decline in visual acuity, it may be possible to delay or prevent the progression of DR. Furthermore, gaining a better understanding of early-stage DR could allow for the development of novel screening strategies.

**Figure 1:**
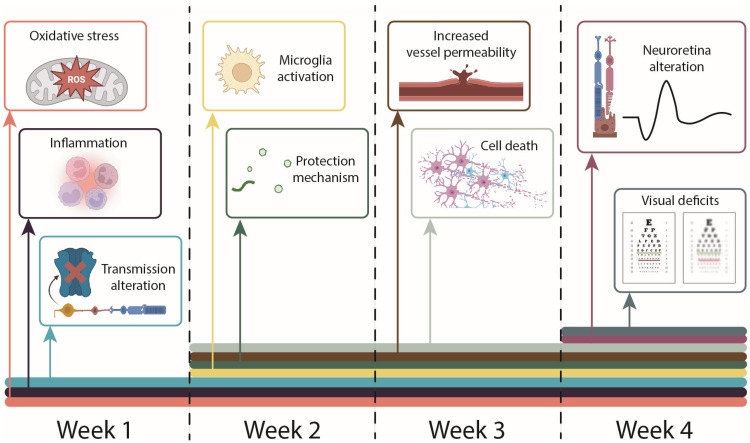
Graphical representation of main events that occur in the streptozotocin-induced rat model of diabetic retinopathy, before onset of vision loss.

The pathophysiology of DR is intricate and multifaceted (Figure 1). Recent research suggests that neuronal changes, attributed to oxidative stress and inflammation induced by hyperglycemia, precede the vascular alterations clinically identified in DR [5]. During the first four weeks of hyperglycemia following the chemical induction of diabetes, rat models exhibit a sequential progression of the disease, including redox imbalance [6,7], activation of microglia [8], secretion of pro-inflammatory cytokines [6,9], neuronal cell death [10], vascular damage [11], neovascularization [12], and decline in visual function [13,14,15].

Based on these findings, the aim of this review is to outline the chronological progression of DR and the key molecular mechanisms involved prior to the decline in visual function. This provides a foundation for future research into targeted therapies. This review also highlights the differences between early and advanced stages and their relevance to translational research. It underscores the need for time-based frameworks in preclinical studies to guide the development of treatments for slowly progressive vision loss.

## 2. Search Strategy

**Figure 2:**
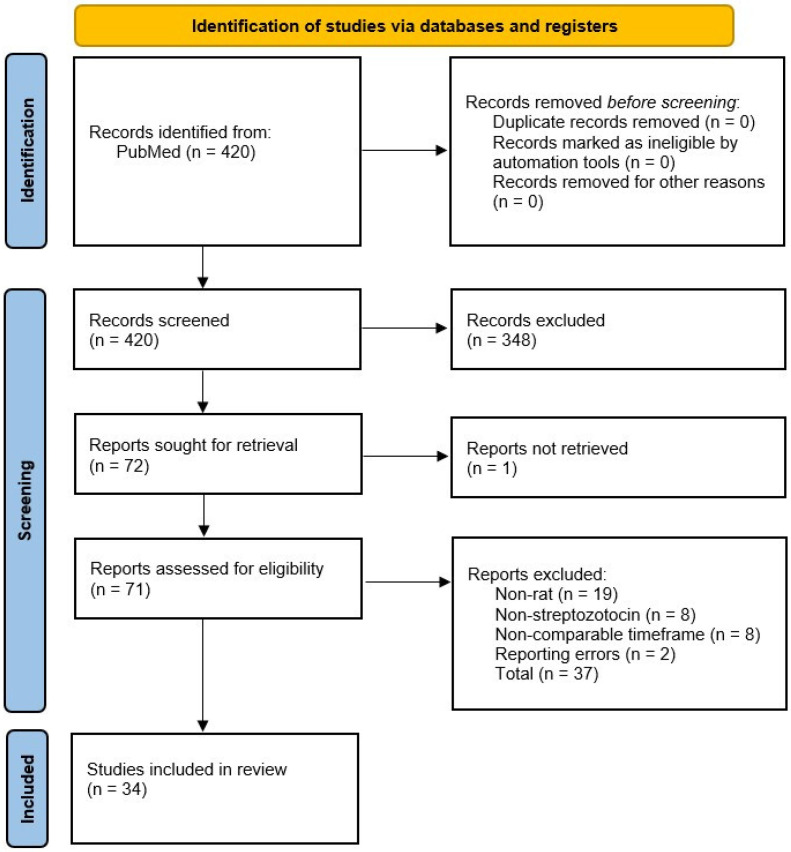
PRISMA flowchart.

The PubMed database was searched for articles from inception up to 26 December 2024. The keywords “rat”, “diabetic retinopathy”, and “streptozotocin” were used to identify relevant articles. The initial search identified 420 articles, of which only 34 met the inclusion and exclusion criteria for the final analysis (Figure 2). Articles were included if they met the following criteria: original research using a rat model; streptozotocin-induced diabetes with stated glycemic thresholds aligning with standard definitions; specified experimental duration and timeframe for observed pathophysiological changes; investigation of disease mechanisms within 28 days of diabetes onset; and clear descriptions of methods used to determine molecular variables. Articles were excluded if they were review articles, commentaries, editorials, or opinion pieces; involved human trials, case studies, or animal models other than rats; used substances other than streptozotocin to induce diabetes (e.g., alloxan, dexamethasone, monosodium glutamate, glucose loading, corticosteroids, or cyclophosphamide); failed to define glycemic thresholds for diabetes diagnosis or used definitions inconsistent with established literature; did not specify experimental duration or timeframe for molecular changes post-diagnosis; examined disease mechanisms beyond 28 days of diabetes onset; or lacked clear methodological descriptions of how variables were measured.

The risk of bias was assessed by two researchers using a modified Systematic Review Centre for Laboratory Animal Experimentation (SYRCLE) tool, with disagreements resolved by a third reviewer [16].

## 3. Chemically Induced Diabetic Rat Model

Animal models that can closely reproduce human disease patterns sit at the centre of preclinical experimentation. However, there is no perfect model, and results from animal testing must only be used as a guide for patient trials [17]. Rodents are used due to their small size, ease of handling, fast reproduction rate, and ease of diabetes induction [4]. Rats are preferred over mice due to their slightly larger weight, which ensures a higher survival rate to the extreme glycemic variations following induction [17]. For the study of diabetes and its complications, there are five methods through which diabetes can be induced: spontaneous, genetic, surgical, diet, and chemical [18]. The spontaneous models develop diabetes without intervention due to genetic predisposition and environmental factors [18]. Examples include the Bio-Breeding (BB) rat, which is limited by a T-cell decrease [18], a modification that does not occur in human diabetes, and the Komeda diabetes-prone (KDP) rat, which overcomes this limitation, but has a low reproductive rate [19] and high cost [20]. Additionally, the generation of diabetes through direct genetic manipulation has created widely employed models, like the Zucker Diabetic Fatty (ZDF) rats [21]. These animals become hyperphagic due to a mutation of the leptin receptor gene, a hormone responsible for appetite suppression [22], making them obese and subsequently leading to type 2 diabetics. This model has been widely employed for the study of advanced complications due to the gradual progression of diabetes [18]. Another genetic model of disease is the Goto-kakizaki (GK) rat, which is limited by difficult pregnancies with few viable offspring [18]. Furthermore, diabetes can be induced by the surgical ligation of pancreatic ducts, or pancreatectomy; however, it is rarely used for the study of diabetic complications due to its traumatic nature [18]. Diabetic models can be created through diet by feeding animals with hypercaloric chow [18]. However, this process is time-consuming, and it is ultimately supplemented by the administration of diabetogenic chemicals [23]. Lastly, there is the induction of diabetes through the administration of chemical, cytotoxic substances—a method that is simple and economically advantageous [18]. The diabetogenic substances commonly used are alloxan and streptozotocin (STZ) [24]. These substances act by binding to the pancreatic β-cell glucose transporter (GLUT-2) and inducting the necrosis of islets [24]. Both substances are limited by the high rate of mortality due to ketosis associated with rapid-onset hyperglycemia [18]. However, STZ is preferred over alloxan due to its longer half-life (one hour compared to two minutes), which assures a more stable solution before administration, fewer fatalities upon induction, and, ultimately, a large literature regarding complications [18]. Moreover, it has been observed that rats develop type 1 diabetes after the administration of lower doses of STZ when compared to mice [4]. In addition to being reliable models for the study of diabetes, rats have been largely employed for the study of DR. Structurally, the retinal elements involved in early DR, neural circuits and vessels, are similar to those of humans. However, it must be noted that rats are nocturnal animals and thus possess a significantly lesser number of cones [25] and no macula [26]. Other differences include a reduced ratio of retinal ganglion cells (RGCs) to photoreceptors and variations in the types of RGCs and amacrine cells present [26].

A key element that must be considered in experimental research is the sex bias [27], as the majority of studies only include male animals. There is a difference in ocular disease incidence in relation to sex, yet the possible relation to sex hormones has not been fully elucidated [28]. In recent decades, research has begun exploring how estrogen, a key hormone for women’s health, influences the progression of this condition. It has been found that estrogen plays a neuroprotective role in retinal health, and its deficiency can contribute to ocular damage, especially during postmenopausal periods [29]. Hao et al. identified two subtypes of estrogen receptors (ERα and ERβ) and showed that ERα is predominantly found in the retina of young women but is rare in men and postmenopausal women. A derivative of estrogen, E2 (17β-estradiol), protects retinal ganglion cells from high glucose damage, and this protection is dependent on the presence of estrogen receptors [29]. Yousefi et al. highlighted that postmenopausal women, who experience a decrease in estrogen levels, are more prone to visual impairment. This study demonstrated that estrogen plays a protective role by increasing retinal blood flow and through its antioxidant actions. Estrogen deficiency, whether due to menopause or ovariectomy, leads to inflammation and oxidative stress, which increases the risk of ocular complications, including diabetic retinopathy [30]. Yamashita et al. used full-field electroretinography to measure retinal function in diabetic rat models. After four weeks of hyperglycemia induced by STZ, a significant decrease in cone response was observed in the ovariectomized group, suggesting that estrogen deficiency contributes to visual impairment in diabetes [31]. In addition to these findings, Schmidl et al. showed that sex hormones, particularly estrogen and progesterone, can influence the progression of DR during pregnancy. Although the risk of progression is higher, women who maintain strict metabolic control during pregnancy do not have an increased risk of retinopathy worsening. However, the risk increases postpartum when metabolic control may be relaxed [32]. In a recent study, Lee et al. found that existing diabetic retinopathy at the onset of pregnancy increased the risk of progression almost tenfold during pregnancy. This emphasizes the need for strict management of diabetes in pregnant women who already have diabetic retinopathy [33].

Additionally, one essential methodological factor is the administration of insulin during diabetes progression. Despite being unreported, insulin is often used to correct hyperglycemic states in diabetic rats. This aspect is of particular interest to DR due to the phenomenon of early worsening. This describes the paradoxical exacerbation of DR after the administration of insulin. Glycemic fluctuations have a greater impact on the progression of DR than constant high glycemic levels, with a pronounced promotion of neovascularization. There is no full explanation for the underlying mechanism, yet a synergistic action of insulin with the vascular endothelial growth factor, secreted by the ischemic retina, is speculated to favour the formation of pathological, hemorrhage-prone vessels [34].

## 4. First Week of Hyperglycemia—Oxidative Stress and Microglia Activation

The initial trigger in the development of DR is hyperglycemia, which activates key pathways of diabetes-related damage (Table 1). These pathways [35] include the polyol, hexosamine, protein kinase C (PKC), advanced glycation end-product formation (AGE), and activation of Poly(ADP-ribose) polymerase. This leads to an overproduction of reactive oxygen species (ROS) beyond the body’s ability to defend itself.

During the first week of hyperglycemia (Table 2), in a STZ induced Long Evans rat, Keap1-Nrf2 levels, the constituents of the main protective pathway against oxidative stressors, were reduced [58].

Thus, the buildup of ROS caused the activation of microglia, cells responsible for the maintenance of retinal homeostasis. In a Sprague Dawley rat model of STZ-induced diabetes, amoeboid state microglia were observed after one week of hyperglycemia [8]. As they activate, local retinal microglia undergo a morphological change. Their cell bodies expand and their processes thicken and shorten [59]. Activated microglia then suffer transcriptional changes and start producing certain types of microRNA (miR) [59]. MicroRNAs, smaller than various other RNA types, possess the ability to bind to messenger RNAs, thereby inhibiting their protein synthesis function [60]. In a Sprague Dawley rat model, Wang et al. described an increase in the levels of microRNA-365 together with a reduction in the metalloproteinase inhibitor 3 (Timp3) protein after one week of hyperglycemia [6]. The abnormality of the miR-365/Timp3 pathway exacerbated oxidative stress within retinal microglia, thus creating a vicious cycle of redox imbalance. Additionally, ROS induce the translocation of the nuclear factor kappa-light-chain-enhancer of activated B cells (NFκB) in the microglia [59]. The levels of NFκB should be further studied to determine the time-point of the initial increase as it physiologically precedes the secretion of cytokines, which were found to be elevated during the first week of hyperglycemia. As a result, activated microglia produce pro-inflammatory cytokines, with a marked increase in TNFα and Il-1β levels [6,9].

The microglial changes observed during the first week are an early event that contributes to neural damage, a decline in cell viability, and ultimately the loss of visual function [59]. Thus, the process of vision loss begins early on, as observed by Morales-Calixto et al. [36], who found that neurotransmission is altered as early as the first week of hyperglycemia. In a Long Evans rat model, they observed an alteration in the number of glycine receptor subunits [36]. These receptors are chloride ion channels responsible for inhibitory neurotransmission. The α2 and α4 subunits form receptor complexes. A reduction in these subunits may disrupt inhibitory signalling in retinal circuits, particularly in RGCs, thereby enhancing excitatory activity and contributing to excitotoxicity. Conversely, an upregulation of the β subunit, which is essential for receptor anchoring, may represent a compensatory mechanism to stabilize receptor function or alter receptor localization, highlighting the need for further investigation into these regulatory changes.

**Table 2:** Molecular findings in streptozotocin-induced rat models of diabetic retinopathy.

	Findings	Author, Year
Week 1		
Oxidative stress		
	↑ miR-365 ↑ miR-221 ↓ TIMP3	Wang, 2018 [6]
	↓ Nrf2 ↓ Keap1	Albert-Garay, 2019 [7]
Inflammation		
	↑ TNFα	Wang, 2018 [6]; Puglia, 2020 [9]
	↑ Il-1β	Wang, 2018 [6]
Neurotransmission alteration		
	↓ Glycine receptor a2, a4 subunits ↑ Glycine receptor b subunit	Morales-Calixto, 2019 [36]
Week 2		
Protection mechanism		
	↑ EPO	Gu, 2019 [41]
Oxidative stress		
	↑ miR-365 ↑ miR-221 ↓ TIMP3	Wang, 2018 [6];
	↑ Nitrotyrosine cells in the INL, GCL, RPE	Dionysopoulou, 2023 [37]
Inflammation		
	↑ TNFα	Dionysopoulou, 2023 [37]; Özay, 2020 [40]; Wang, 2018 [6]
	↑ B1 receptor	Hachana, 2018 [38]
	↑ INF-gamma	Özay, 2020 [40]
	↑ c-myc	Zhang, 2019 [61]
	↑ Il-1β	Wang, 2018 [6]
Microglia activation		
	↑ Iba-1	Dionysopoulou, 2023 [37]
	↑ Amoeboid morphology	Hachana, 2018 [38]
	↑ iCAM-1	Shi, 2021 [8]
Vessel permeability		
	↑ VEGF	Dionysopoulou, 2023 [37]
	↑ VEGF-A ↑ VEGFR-2	Hachana, 2018 [38]
	↑ Phosphorylated VE-cadherin	Liu, 2020 [39]
	↑ Evans Blue extravasation	Hachana, 2018 [38]
Cell death		
	↑ MMP-2, MMP-9	Özay, 2020 [40]
	↓ PERG	Dionysopoulou, 2023 [37]
	↓ GCL and IPL thickness	Dionysopoulou, 2023 [37]
Week 3		
Protection mechanism		
	↑ HO-1	Giunta, 2023 [42]
Oxidative stress		
	↓ Nrf2	Albert-Garay, 2021 [7]
	↑ G-6-P ↑ Glycogen ↑ Lactate	Ramírez-Pérez, 2020 [43]
Inflammation		
	↑ COX-2 ↑ iNOS	Giunta, 2023 [42]
Week 4		
Protection mechanism		
	↑ EPO ↑ EPOR	Giunta, 2023 [42]
Oxidative stress		
	↓ SOD ↑ iPF2a	Fathalipour, 2019 [45]
	↑ ROS ↑ Nrf2 ↑ HO-1	Canovai, 2022 [14]
	↓ Nrf2, NQO1, HO-q ↑ Keap1 ↑ MDA ↓ SOD ↓ CAT ↓ GPx	Shi, 2020 [12]
Inflammation		
	↑ IL-1β	Bai, 2021 [44]; Zhang, 2019 [61]; Ibán-Arias, 2018 [54]
	↑ Il-18	Bai, 2021 [44]
	↑ Il-6	Canovai, 2022 [14]; Clapp, 2019 [56]; Zhang, 2019 [61]
	↑ HIF-1a ↑ ANGPTL4	Yang, 2019 [48]
	↑ NFκB	Canovai, 2022 [14]; Shi, 2020 [12]
	↑ SOX9	Li, 2023 [47]
	↓ FKN	Jiang, 2022 [51]
	↑ TNFα	Shi, 2020 [12]; Ibán-Arias, 2018 [54]; Zhang, 2019 [61]
	↑ MMP-2 ↓ IL-10 ↓ TIMP-1	Shi, 2020 [12]
	↑ c-myc	Zhang, 2019 [61]
	↓ Iba-1	Shi, 2021 [8]
Microglia activation		
	↑ SOX9 ↑ TXNIP	Li, 2023 [47]
	↑ GFAP	Li, 2023 [47]; Canovai, 2022 [14]; Zhang, 2018 [53]; Ibán-Arias, 2018 [54]; Gu, 2019 [41]
Vessel permeability		
	↑ HIF-1α	Canovai, 2022 [14]; Yang, 2019 [48]; Gu, 2019 [41]
	↑ VEGF	Zhang, 2018 [53]; Canovai, 2022 [14]; Gu, 2019 [41]; Clapp, 2019 [56]
	↑ Evans Blue extravasation	Canovai, 2022 [14]; Clapp, 2019 [56]
	↑ ANGPTL4	Yang, 2019 [48]
	↑ Vessel formation in IPL	Shi, 2020 [12]
	↓ miR29a ↓ miR-29b	Zhang, 2018 [53]
	↓ MEG3	He, 2021 [55]
	↑ Dilated tortuous vessels ↑ hemorrhage	Fu, 2021 [11]
Cell death		
	↓ RGCs	Fathalipour, 2019 [45]
	↑ Condensed nuclei in GCL	Shi, 2020 [12]
	↓ Glutamine synthase	Zhang, 2018 [53]; Gu, 2019 [41]
	↓ GLAST	Gu, 2019 [41]
	↓ β-III tubulin	Ma, 2018 [10]
	↓ Cell viability	Bai, 2021 [44]
	↑ TUNEL-positive cells	Bai, 2021 [44]; Ma, 2018 [10]; Ibán-Arias, 2018 [54]
	↓ TH protein	Ma, 2018 [10]
	↑ Caspase 3	Canovai, 2022 [14]; Ma, 2018 [10]
	↑ ONL cell death	Jiang, 2022 [51]
	↑ p75NTR	Ibán-Arias, 2018 [45]
	↓ ONL, INL thickness	Bai, 2021 [44]
	↑ Degenerate capillaries	Bai, 2021 [44]
	↓ Retinal thickness	Fathalipour, 2019 [45]; Li, 2023 [43]; Fu, 2021 [11]
	No MMP level alteration	Şahin, 2021 [57]
Neuroretinal alteration		
	↓ Electroretinogram a-wave, b-wave amplitude	Naderi, 2019 [13]; Canovai, 2022 [14]
	↓ CNTF protein	Ma, 2018 [10]
	↑ p-ERK	Fathalipour, 2019 [45]; Ibán-Arias, 2018 [45]
	↓ p-AKT	Fathalipour, 2019 [36]
	↓ Uptake of [^18^F]FP-(+)-DTBZ ↓ VMAT2	Li, 2020 [49]
	↓ NFL-, bNOS-, and TH-IRs	Ibán-Arias, 2019 [50]; Ibán-Arias, 2018 [45]
Visual function deficits		
	↓ Spatial frequency thresholds ↓ Contrast sensitivity	Allen, 2018 [15]

↑ increase, ↓ decrease.

Similar pathophysiological mechanisms have been observed in human trials. Patients with early-stage diabetic retinopathy, specifically non-proliferative, exhibit alterations in oxidative stress [43]. Lipid peroxidation levels are elevated, with increased serum concentrations of malondialdehyde [43,62,63]. In addition, nitrosative stress is increased, indicated by elevated nitrite/nitrate levels due to the conversion of nitric oxide into peroxynitrite, which has been found to damage the blood–retinal barrier [43]. Key antioxidant enzymes, such as catalase and glutathione peroxidase, show dysregulated activity [43]. Furthermore, based on the understanding that microglia contribute to inflammation in early diabetic retinopathy, a randomized, double-blind clinical trial was conducted to test the hypothesis that a microglia-centred therapy could slow the progression of the disease [64]. The study concluded that doxycycline could have varying effects based on the stage of retinopathy [64].

## 5. Second Week of Hyperglycemia—Increased Vessel Permeability

Throughout the second week of hyperglycemia, the state of oxidative stress imbalance persisted. Dionysopoulou et al. noted an increase in the number of nitrotyrosine-positive cells within the retina of a Sprague Dawley rat model with STZ-induced hyperglycemia [37]. Nitrotyrosine is a marker of neurodegeneration caused by oxidative stress, which reflects the nitration of neuronal cytoskeleton proteins by ROS [65]. In response to the constant threat of redox imbalance, the body activates certain protective mechanisms. It has been stipulated that one such protective measure is the increase in the synthesis of erythropoietin (EPO) within the retina [66]. EPO has a dual role in DR, being protective in the early stages and pathogenic in the proliferative stages [66]. In early DR, EPO may stabilize retinal vasculature and provide neuroprotective and anti-inflammatory effects [66]. However, in proliferative diabetic retinopathy (PDR), EPO contributes to neovascularization through angiogenic mechanisms similar to VEGF, with elevated levels observed in vitreous PDR patients [66]. Identifying the optimal timing for EPO-based interventions remains an important direction for future research [66]. Thus, in their study, Gu et al. found an increase in the levels of EPO after two weeks of hyperglycemia in a Sprague Dawley rat model [30].

Furthermore, the pro-inflammatory state persisted during the second week of DR development, with high levels of TNFα [37], Il-1β [6], and, additionally, increased IFN-γ [40]. Moreover, Zhang et al. identified c-myc [61] as a modulator of the production of inflammatory cytokines by activated microglia. Likewise, Hachana et al. observed an increased expression of the bradykinin B1 receptor in the retina of diabetic Wistar albino rats [38]. This receptor is induced by oxidative stress or inflammation and is highly expressed in the retinas of diabetic patients [38]. In addition to the production of inflammatory cytokines, the continuously activated microglia [37,38] secreted intracellular adhesion molecule 1 (ICAM-1) [12], an adhesion protein involved with leukocyte recruitment [67]. This causes an accumulation of leukocytes and eventual occlusion of retinal vessels, a phenomenon referred to as leukostasis [67].

Hypoxia is the primary factor underlying the downstream modifications observed in this context. When combined with constant hyperglycemia, it significantly promotes the upregulation of vascular endothelial growth factor (VEGF) [37,38]. Cells that are put in a hypoxic environment generate hypoxia-inducing factor (HIF)-1α, a transcription factor that triggers the release of VEGF. In DR, the inhibition of HIF-1α suppresses VEGF expression and slows angiogenesis over time [68]. Further research is required to evaluate early HIF-1α levels, as early as the second week of hyperglycemia, since VEGF is already elevated at that stage. The high level of VEGF increases vascular permeability [38] by the endocytosis [69] of vascular endothelial (VE)-cadherin [39], a primary adhesive junction between cells in the endothelium of blood vessels [70]. In addition to the increase in permeability, Özay et al. found, in a Wistar albino rat model of diabetes, high levels of matrix metalloproteinases, MMP-2 and MMP-9 [40]. Matrix metalloproteinases are enzymes involved with the degradation of the extracellular matrix, which is further linked to angiogenesis. Their enzymatic activity is counterbalanced by tissue inhibitors (TIMPs). Thus, an imbalance between MMPs and TIMPs has been noted in individuals with diabetes [71]. Similarly, Shi et al. [12] observed an increase in levels of MMP-2 coupled with a decrease in TIMP-1 activity. However, controversial results have been reported regarding MMPs, with a study by Şahin et al. finding no significant changes even after four weeks of diabetes [57]. Further research is needed to clarify the precise time frame in which MMP levels begin to change.

Prior to observable vascular injury, the effects of oxidative stress, inflammation, and hypoxia cause notable neural alterations. Early on, there is a decrease in the viability of retinal ganglion cells [37], which are crucial for processing light and whose cellular death results in vision impairment.

## 6. Third Week of Hyperglycemia—Progression of Redox and Inflammatory Imbalances

There is limited data available regarding the third week of hyperglycemia following diabetes induction. However, there is a notable rise in a crucial molecule following three weeks of hyperglycemia: lactate [43]. Lactate, serving as a marker of redox imbalance, exhibits a notable increase specific to the third week, without prior elevation. Additionally, Giunta et al. found an increase in the levels of inducible nitric oxide synthase (iNOS) and cyclooxygenase-2 (COX-2), primary drivers of inflammation [42]. Moreover, they noted higher heme oxygenase 1 (HO-1), an inducible enzyme that plays a central role in cell defence by breaking down heme into the antioxidant, anti-inflammatory, and cytoprotective biliverdin and its metabolite, bilirubin [72].

## 7. Fourth Week of Hyperglycemia—Visual Deficits

In addition to the previously described alterations, the fourth week of hyperglycemia is characterized by significant vessel structural modifications and neural degeneration, leading to the ultimate loss of visual function. Thus, upon fundus examination, retinal vessels become dilated and tortuous, with areas of hemorrhage [11], capillary degeneration [44], and first instances of neovascularization [12]. Moreover, He et al. [55] observed using transmission electron microscopy the partial expansion of retinal vessels. In accordance with these findings, there are biochemical changes that promote vessel formation. He et al. described a decrease in the maternally expressed gene 3 (MEG3), which has anti-angiogenetic properties. Similarly, Yang et al. described high levels of angiopoietin-like protein 4 (ANGPTL4), which favours neovascularization. Additionally, the activation of microglia [14,41,47,53,54], together with cell death, are the main components of neural degeneration, precursory of visual impairment [73]. There is a decrease in cellularity within the retina, with lower thickness of the ganglion cell layer (GCL) [44], inner nuclear layer (INL) [44], and outer nuclear layer (ONL) [51]. Overall, the entire retinal thickness is decreased [11,45,47], with a higher expression of the apoptosis marker, caspase-3 [14]. Thus, studies have identified multiple types of cellular death during the progression of DR [74]. For neural cell death, the main mechanism is believed to be apoptosis, as shown by increased staining through the TUNEL (Terminal dUTP Nick End Labelling) assay [10,44,54]. This staining is applied to paraffin-embedded samples and it can detect broken DNA fragments, which form at the end of apoptosis [75]. Additionally, there is a marked decrease in ciliary neurotrophic factor [10], a primary protective cytokine of the retina [76]. Ganglion cell death is marked by a decrease in β-III-tubulin [10], a fundamental element of neuronal microtubules [77]. Additionally, the death of amacrine cells, the source of retinal dopamine, is denoted by a decrease in tyrosine hydroxylase [10]. This enzyme catalyzes the conversion of tyrosine, an amino acid, into dopamine, representing the pivotal step in dopamine synthesis. Furthermore, injury to the amacrine cells has been detected using positron emission tomography by employing a tracer that also serves as a biomarker for dopamine presynaptic vesicles [49]. The degeneration of neural cells could be, in part, related to the impairment of the glutamate–glutamine cycle in the retinal glial cells. Glutamate plays a paradoxical role in the retina—whilst it is the key neurotransmitter of the visual pathway, and, in high concentrations, it can become toxic and cause neural degeneration. Thus, its concentration is maintained through uptake into the glial cells by the glutamate transporter, glutamate–aspartate transporter (GLAST), and metabolization by glutamine synthetase [78]. Ultimately, reduced levels of glutamine synthetase [41,53] and/or GLAST have been linked to a fast decrease in visual acuity [78]. Additionally, studies have speculated that pyroptosis may play a role in the progression of DR [74]. Pyroptosis is a type of cell death [79] initiated by the formation of an inflammasome [80], a scaffold that serves to recruit and activate caspase-1. This enzyme then enables the release of pro-inflammatory cytokines, such as IL-1β. Thus, in a late-stage diabetic rodent model, the activation of the caspase-1/IL-1β pathway was observed in retinal glial cells [74]. The activation of this pathway in early diabetes has not been fully elucidated; however, several studies [44,54,61] observed an increase in IL-1β levels after four weeks of hyperglycemia.

After four weeks of hyperglycemia, the STZ-induced diabetic rat model shows notable changes in visual function, which are documented through electroretinograms (ERGs) [14]. An ERG is a non-invasive technique used to assess light perception in rodents [13]. The scotopic ERG measures rod cell function in dim light, while the photopic ERG evaluates cone cell activity under bright light [81]. The Photopic Negative Response (PhNR), a part of the photopic ERG, reflects the activity of retinal ganglion cells that transmit visual signals to the brain [81]. In their study, Naderi et al. report a decrease in scotopic ERG amplitudes in diabetic rats one month after the onset of diabetes [13]. Similarly, Canovai et al. observe a reduction in scotopic ERG amplitudes one month after diabetes diagnosis, without histological changes in the retinal nerve layers [14]. Lee et al. also found a gradual amplitude reduction in ERG recordings, particularly in scotopic and PhNR responses, with less significant changes in the photopic response [81].

## 8. Conclusions

Studies on STZ-induced diabetic rats show a sequential progression of diabetic retinopathy (DR) over four weeks of hyperglycemia. Before vision loss, morphofunctional changes occur, including redox imbalance, microglial activation, and inflammation in week one, followed by neuronal cell death and increased vascular permeability in week two. In week three, redox imbalance and inflammation worsen, and by week four, vessels dilate, become tortuous, neovascularization occurs, and retinal thickness decreases, leading to vision loss.

Hyperglycemia activates pathways that lead to excessive ROS production, overwhelming the body’s defence mechanisms. This results in reduced Keap1-Nrf2 levels in the first week, triggering microglial activation and increasing pro-inflammatory cytokine production. Microglia undergo morphological and transcriptional changes, contributing to neuronal damage.

Neurotransmission is altered in week one, with decreased glycine receptor subunits affecting inhibitory signalling, leading to excitotoxicity. In week two, hypoxia from blood vessel occlusion increases VEGF, raising vascular permeability and contributing to early retinal damage. Estrogen plays a neuroprotective role in retinal health, and postmenopausal women with low estrogen levels are more susceptible to vision issues. Insulin treatment in diabetic rats can paradoxically worsen DR by exacerbating blood glucose fluctuations, which impact DR progression more than consistently high glucose levels.

## Figures and Tables

**Table 1:** Methodological characteristics of the diabetic retinopathy studies, including rat model, sex, age, initial weight, streptozotocin scheme, cut-off glycemia, days and weeks since diagnosis of diabetes until sample collection, and final glycemia.

Rat Model	Sex	Age (Weeks)	Initial Weight (g)	STZ Scheme	Cut-Off Glycemia (mg/dL)	Time from Diagnosis (days)	Time from Diagnosis (weeks)	Final Glycemia (mg/dL)	Author, Year
									Week 1
Sprague Dawley	male	NA	150	1 dose ip 60 mg/kg	250	7	1	NA	Wang, 2018 [6]
Long Evans	female	8	179 ± 7	1 dose ip 98/kg	250	7	1	355 ± 14	Albert-Garay, 2021 [7]
Sprague Dawley	male	NA	175–200	1 dose iv 60 mg/kg	250	10	1	NA	Puglia, 2020 [9]
Sprague Dawley	male	NA	120	1 dose ip 60 mg/kg	300	7	1	544 ± 14	Shi, 2021 [8]
Long Evans	female	NA	180–200	1 dose ip 90 mg/kg	250	7	1	382 ± 18	Morales-Calixto, 2019 [36]
									Week 2
Sprague Dawley	male, female	NA	200–300	1 dose ip 70 mg/kg	300	14	2	NA	Dionysopoulou, 2023 [37]
Wistar albino	NA	6–8	200–250	1 dose ip 65 mg/kg	360	14	2	480 ± 34	Hachana, 2018 [38]
Sprague Dawley	male	NA	130–160	1 dose ip 60 mg/kg	300	14	2	NA	Liu, 2020 [39]
Wistar albino	male	12–16	180–240	1 dose ip 45 mg/kg	250	14	2	NA	Özay, 2020 [40]
Sprague Dawley	male	NA	180	1 dose ip 60 mg/kg	250	14	2	NA	Gu, 2019 [41]
									Week 3
Sprague Dawley	male	NA	200–250	1 dose ip 60 mg/kg	250	21	3	312 ± 28	Giunta, 2023 [42]
Long Evans	female	8	170 ± 15	1 dose ip 98/kg	250	20	3	483 ± 15	Albert-Garay, 2021 [7]
Long Evans	NA	NA		1 dose ip 98 mg/kg	250	20	3	NA	Ramírez-Pérez, 2020 [43]
Long Evans	female	NA	180–200	1 dose ip 90 mg/kg	250	21	3	480 ± 15	Morales-Calixto, 2019 [36]
									Week 4
Wistar albino	male	NA	270–300	1 dose ip 55 mg/kg	300	28	4	454 ± 56	Naderi, 2019 [13]
Wistar albino	male	NA	220–280	1 dose ip 60 mg/kg	300	28	4	324 ± 18	Bai, 2021 [44]
Wistar albino	male	NA	250–300	3 dose ip 65 mg/kg	300	28	4	NA	Ma, 2018 [10]
Sprague Dawley	male	NA	200–225	1 dose ip 60 mg/kg	300	28	4	494 ± 21	Fathalipour, 2019 [45]
Wistar albino	male	9	NA	1 dose ip 60 mg/kg	250	28	4	NA	Kida, 2019 [46]
Sprague Dawley	male	NA	NA	1 dose ip 65 mg/kg	360	28	4	396 ± 36	Li, 2023 [47]
Sprague Dawley	male	8	180–220	1 dose ip 65 mg/kg	300	28	4	504 ± 18	Yang, 2019 [48]
Sprague Dawley	male	8	130–160	1 dose ip 65 mg/kg	300	28	4	468 ± 36	Li, 2020 [49]
Sprague Dawley	male, female	NA	180–300	1 dose ip 70 mg/kg	350	28	4	NA	Ibán-Arias, 2019 [50]
Sprague Dawley	male	8	200	1 dose ip 65 mg/kg	250	30	4	550±10	Canovai, 2020 [14]
Sprague Dawley	male	NA	120–160	1 dose ip 60 mg/kg	300	28	4	502 ± 21	Jiang, 2022 [51]
Wistar albino	male	NA		1 dose ip 55 mg/kg	450	28	4	540 ± 36	Shi, 2020 [12]
Sprague Dawley	male	7–8	200–300	1 dose ip 60 mg/kg	350	28	4	590	Jung, 2022 [52]
Sprague Dawley	male	4–6		1 dose ip 60 mg/kg	300	28	4	NA	Zhang, 2018 [53]
Long Evans	male	9	325–350	1 dose ip 100 mg/kg	250	28	4	NA	Allen, 2018 [15]
Sprague Dawley	male, female	NA	180–300	1 dose ip 70 mg/kg	350	28	4	NA	Ibán-Arias, 2018 [54]
Sprague Dawley	male	6–7	260–360	1 dose ip 60 mg/kg	300	30	4	NA	He, 2021 [55]
Sprague Dawley	male	6–8	180–220	1 dose ip 60 mg/kg	300	30	4	400 ± 30	Fu, 2021 [11]
Wistar albino	male	NA	150–180	1 dose ip 60 mg/kg	250	30	4	NA	Clapp, 2019 [56]
Sprague Dawley	male	28	250 ± 50	1 dose ip 35 mg/kg	250	28	4	NA	Şahin, 2021 [57]

## Data Availability

No new data were created or analyzed in this study. Data sharing is not applicable to this article.
